# Role and responsibilities of a forensic mental health nurse: a scoping review protocol

**DOI:** 10.1136/bmjopen-2025-098745

**Published:** 2025-07-15

**Authors:** Lindsay Tulloch, EIleen Harkess-Murphy, Helen Walker, Joshua Cheyne, Marie McCaig, Robin Ion

**Affiliations:** 1 PhD Student, University of the West of Scotland, Hamilton, UK; 2 Health and Life Sciences, University of the West of Scotland, Paisley, UK; 3 Forensic Network, Lanarkshire, UK; 4 University of the West of Scotland, Paisley, UK; 5 School of Health & Life Sciences, University of the West of Scotland, Dumfries, UK; 6 Mental Health Nursing, Independent Scholar, Dundee, UK

**Keywords:** Nursing research, Forensic psychiatry, Vulnerable Populations, MENTAL HEALTH

## Abstract

**Abstract:**

**Introduction:**

Forensic mental health nursing (FMHN) is a subspeciality of psychiatric nursing. An area of mental health nursing care that is situated at the intersection of health, social and criminal justice systems. Over the past two decades, FMHN has evolved beyond custodial and containment practice. Contemporary FMHN has an emphasis on therapeutic interventions, identifying patients as partners in care and nursed through a trauma-informed, recovery-orientated lens. Numerous scholars have examined the role of the FMHN and its inherent complexities. However, much of the existing literature is outdated and is limited in scope, describing the role and responsibilities of an FMHN relevant to contemporary practice. This paper maps the literature over the last 20 years to establish what explicitly defines the modern FMHN, specifically examining factors that have shaped the role and influenced patient outcomes and care delivery; including areas of good practice.

**Methods and analysis:**

In line with the Joanna Briggs Institute (JBI) guidance on scoping reviews, including Arksey and O’Malley’s (2005) five-step framework, we will conduct a search within MEDLINE (EBSCO), CINAHL (EBSCO), PsycINFO (Ovid) and the Cochrane Database of Systematic Reviews and Cochrane Central Register of Controlled Trials in the Cochrane Library. The first author conducted a preliminary search in October 2024 to identify literature in this area and a review of keywords to develop the foundation search strategy. The search strategy was constructed in the MEDLINE (EBSCO) database May 2025 by the lead author and an information specialist/librarian. Eligibility criteria of publications written in English, with a date range of 2004–2024, including the first quarter of 2025, forensic mental health nurse population and secure inpatient settings. All extracted literature will be exported into EndNote V.21 in order to support the removal of duplicates and assist in the screening and selection process. A two-step data selection process will include stage one, where two authors independently conduct a preliminary title and abstract screen of all extracted data using a data extraction instrument developed per JBI scoping review guidance. Each paper will be categorised as ‘yes’, ‘no’ or ‘maybe’. Step two: All documents categorised as ‘yes’ or ‘maybe’ will undergo a full-text screening. Narrative summaries and tables will present the results in full.

**Ethics and dissemination:**

This scoping review will analyse existing published data; therefore, ethical approval is not required. The findings of this review will be presented at local and international conferences and published in peer-reviewed journals. The formal search will commence in June 2025, with an aim to submit in full for peer-review publication by October 2025.

**STRENGTHS AND LIMITATIONS OF THIS STUDY**

- This scoping review protocol has been registered with the Open Science Framework.
- A multiprofessional group consisting of forensic mental health professionals, researchers and educators collaborated with an experienced academic information specialist/librarian to develop the search strategy.
- Conducting a scoping review to explore the available literature worldwide allows for the identification of variations in role definition.
- Studies not published in English may result in the exclusion of relevant literature.
- Studies published before 2004 may exclude significant findings.

## Introduction

### What is forensic mental health

 Forensic mental health nursing (FMHN) is a subspeciality of psychiatric nursing. An area of mental health nursing care that is situated at the intersection of health, social and criminal justice systems. FMHN care consists of registered mental health nurses who practice across a variety of forensic settings such as secure inpatient hospitals, prisons, courts and police custody, including the community. People under the care of forensic mental health services have typically been charged with a criminal offence, remanded or committed to custody for assessment and/or treatment for their mental illness. By law, they are required to receive specialist care due to their mental illness and their potential risk to the health and safety of themselves and/or others.^1^

The Mental Health (Care and Treatment) (Scotland) Act 2003, guided by the Milan Principles, shifted the focus of FMHN care from a custodial and containment model to one that remains to this day, and is rooted in a rights-based, person-centred and recovery-focused care.^2 3^ As a consequence, there has been an emphasis placed over the last two decades on legislative human rights and ethical practices in care delivery with a promotion of least restrictive practices and increased patient and carer involvement.^4^ The Act influenced a culture change from containment and risk aversion towards evidence-based, risk formulation, recovery-focused care practices.

FMHNs perform a wide range of clinical, rehabilitative therapeutic care to people with severe and enduring mental health needs. They play a critical and complex role, providing care for a complex, fragile patient population, with relational security challenges. Relational security is a key component in forensic services. It refers to the quality of relationships between staff and patients (also known as mentally disordered offenders). These relationships are centred on the therapeutic connection that generates trust, openness and respect, as opposed to relying on the procedural measures and controlled environment such as locked wards, searching and constant observation. In essence, the quality of the therapeutic relationship, along with gaining an understanding of the mentally disordered offenders’ risks and triggers, is a fundamental feature to maintaining a safe environment.

There is a reported ethical tension of FMHNs being forced into a dual role to preserve safety and practice with care and compassion. As a consequence, the role of an FMHN requires continuous adaptations to meet the needs of this unique population, while ensuring patient, staff and public safety and the delivery of therapeutic care.^5^ As a consequence, the daily role of an FMHN will often include the management of violence and aggression, and at times, the implementation of interventions to ensure the protection and safety of self, others and the public is maintained.^6^

This often necessitates the use of restrictive practices such as emergency chemical interventions, physical restraint and environmental restraint, such as seclusion, which, as a result, present their own unique challenges. As a result, FMHNs must therefore navigate a dual role; they are both clinicians and custodians. Building therapeutic, trusting relationships, while enforcing boundaries, and when necessary, applying restrictive practices as a last resort.^7^ The management of violence and aggression is reported to be a daily ethical tension for FMHN, and as a consequence, they often experience a higher risk of burnout, stress and work-related trauma.^7^,^9^

Forensic mental health patients present with complex and challenging needs that require specialist care. Therefore, FMHNs must navigate a delicate line of care for individuals living with severe and enduring mental health conditions, who often experience a dual stigmatisation of mental illness and offending behaviour.^10^

The growth and development of this subspeciality is reflected in the contemporary delivery of practice, evidence-based risk management and trauma-informed practices. In addition to the expanding scope of the workforce, practice is extending to low, medium and high secure inpatient settings, prisons, courts and community settings with a number of developing specialist FMHN roles; consultant nurses, clinical nurse specialists and researchers.^11 12^

This advancement over the last two decades is a result of a greater understanding of a population of people engaged in criminal justice systems, many of whom predominantly experience comorbidity of conditions, substance misuse, untreated and misdiagnosed mental health disorders, including trauma and complex personality disorders.^13^

### Knowledge gap

FMHN has been practised within the UK for almost two centuries, with the first of four high secure FMH hospitals opening its doors in 1863.^14^ Little was known about FMHN until the late 20th century, with most of the literature being documented from the 1990s.^15^ Since then, many scholars have published literature in an attempt to support practitioners, managers and leaders on role definition and an explanation of its complexities.^16^,^18^ However, much of the existing literature is almost two decades old.^14^ The most recent publications by Martin and colleagues in 2013, which outlined FMHN Standards of Practice and a systematic review by Sorumluluklari and colleague in 2017. The systematic review highlighted an international ambiguity and conflict surrounding the role and responsibilities of a forensic psychiatric nurse. Since these publications, much of the literature has been focused on its complexities, such as the management of violence and aggression and restrictive practice use, recruitment and retention in FMHN.^6^,^817^ There is a notable dearth of international published literature in the last two decades exclusively depicting the contemporary role and responsibilities of a FMHN.

### Educational gap

In 2018, the Nursing and Midwifery Council introduced the ‘Future Nurse’ standards, revising its preregistration nurse education course. This change led to concerns about the dilution of mental health specialisation and the beginning of a more generic nurse education.^19 20^ This change presents significant implications for the future of the role of an FMHN. Without the foundations of mental health nursing education being taught at undergraduate level, this will leave newly qualified registrants ill-equipped to deal with the complex presentation of mentally disordered offenders in forensic mental health. It has therefore never been more timely to scope out a contemporary international understanding of the unique features of an FMHN's role and their impact on patient outcomes and care delivery.

A scoping review is particularly suitable for defining the international role and responsibilities of an FMHN; it allows for a comprehensive mapping of existing literature across the field. The landscape of FMH is diverse and fragmented by nature. There are differing levels of secure settings, patient populations and professional and clinical care practices. This makes it challenging due to its heterogeneous dimensions and sources.

Conducting a scoping review is of particular importance now, and necessary given the legislative changes that have reshaped how and where mentally disordered offenders receive care and treatment for their mental illness. These shifts have particular implications for clinical practice and policy development. The expansion to the workforce and changes to undergraduate training present a timely opportunity to examine how the profession has evolved over the past two decades. By scoping a wide range of existing published literature, the authors are able to identify emerging trends and highlight research gaps, providing valuable insights into the developments of the role and responsibilities across a diverse and international landscape.

### Objectives and review questions

The objective of this scoping review is to establish the extent of the literature to provide an overview of the role and responsibilities of a forensic mental health nurse:

1. What are the primary roles and responsibilities of a registered mental health nurse practising in a forensic setting?
2. What currently defines a forensic mental health nurse?
3. What is known about the changes in the professional standards of practice influencing the role of the forensic mental health nurse?
4. What evidence is there of good practice being identified?

### Methods and analysis

This scoping review will adopt the Joanna Briggs Institute (JBI) guidance on scoping reviews, including Arksey and O’Malley’s (2005) five-step framework, which is a systematic approach to mapping literature concepts, evidence and gaps.^21^,^23^ Both frameworks have been reported to enhance the rigour, transparency and trustworthiness of scoping reviews.^24 25^

The formal search will commence in May 2025, with an aim to submit in full for peer-review publication by Autumn 2025. The protocol has been registered with the Open Science Framework (OSF).^26^

### Eligibility criteria

The eligibility criteria will be based on the JBI; population, concepts and context elements model,^25 27^ including the types of evidence sources:

1. Participants: registered mental health nurses inclusive of any gender, age, grade, experience and country.
2. Concept: role and responsibilities of a mental health nurse practicing within a forensic mental health setting.
3. Context: adult forensic mental health, secure inpatient, court, prison, practice, training, development and educational settings including leadership and management.
4. Types of evidence sources: empirical research, full text publications, research paper, thesis, consisting of a selection of research designs that include quantitative, qualitative and mixed methods.
5. Publication criteria date range between 2004 and 2024 and the first quarter of 2025.
6. No restriction on geographical region.

The following exclusion criteria will be applied:

1. Research conducted not in a forensic mental health specialty.
2. Research conducted in non-adult forensic mental health settings, such as child, adolescent or geriatric forensic mental health settings.
3. Research conducted in forensic intellectual disability and/or autism specialty population and/or settings.
4. Research not conducted in a secure inpatient setting.
5. Research conducted on forensic mental health professionals other than registered mental health nurses practicing in a forensic mental health setting, this includes enrolled nurses, associate nurse practitioners and healthcare support workers.
6. Opinion, debate, protocols, discussion or review papers.
7. Published before the year 2004.
8. Papers that are not published in English.

### Information sources

The information sources being used include:

1. We will conduct exhaustive searches of relevant sources to a comprehensive literature search for evidence-based practice: MEDLINE (EBSCO), CINAHL (EBSCO; Cumulative Index to Nursing and Allied Health Literature), the Cochrane Database for Systematic Reviews, that provides peer reviewed systematic reviews and the Cochrane Central Register of Controlled Trials in the Cochrane Library that provides a wide range of randomised and controlled trials, PsycINFO (Ovid) and the British Library EThOS e-theses online.
2. A review of reference lists and citing articles of all included sources will be screened for additional published articles relevant, including cited reference tracking using Google Scholar (scholar.google.co.uk/), Cited Reference Search and citation chaser (estech.shinyapps.io/citationchaser/).^28^
3. Publication criteria date range between 2004 and 2024 and the first quarter of 2025.

### Search strategy

This search strategy was constructed and tested in the MEDLINE (EBSCO) database during October and November 2024 by an information specialist/librarian and the lead author (online supplemental appendix 1). It is designed to retrieve records pertaining to the role and responsibilities of a forensic mental health nurse. It is constructed using a combination of Medical Subject Headings and uncontrolled vocabulary/keywords.

In keeping with the Preferred Reporting Items for Systematic Reviews and Meta-Analyses extension for scoping reviews (PRISMA-ScR) guidance checklist, the developed search strategy has been developed for one electronic database and prepared to replicate across all additional databases selected for this review.^27 29^

This search will be used as the basis for all subsequent databases included in this review.

### Data extraction process and evidence selection

All extracted literature will be exported into EndNote V.21 in order to support the removal of duplicates, screening and selection process.

The two-step process will include: two authors (LT and HW) in stage one, independently conducting a preliminary title and abstract screening of all extracted data, using a data extraction instrument developed in accordance with the JBI guidance and presented in online supplemental appendix 2. Each paper will be categorised as ‘yes’, ‘no’ or ‘maybe’. Step two will include all papers that were categorised ‘yes’ or ‘maybe’ undergoing a full-text screening by authors LT, HW, EH-M and MM. Reviewer comments will be provided on each paper, including those excluded from the review and also recorded in the data extraction instrument.^25 27 29^

Any conflicts that emerge between the reviewers at each step of the selection process will be handled through discussion with the two additional authors LT and RI, with a consensus being reached.

The data extraction instrument will be piloted on a small sample of the data to ensure it captures all of the necessary elements of the data. The tool will be revised and refined as required to ensure it is fit for purpose.

### Data analysis and presentation of results

Qualitative findings will be synthesised using a thematic analysis approach by authors LT, HW, EH-M and MM. This will involve the identification, analysis and reporting of themes focusing on the role and responsibilities of a forensic mental health nurse, as well as the influencing factors related to the professional standards, and their impact and outcomes.^24 25^

Quantitative data extracted from the search will be reported using frequency counts and visually presented into tables or figures by authors LT, HW, EH-M and MM. These will detail the study types, aims, methodology, key findings, including role and responsibilities definition of a forensic mental health nurse and the influencing factors of professional standards, their impact and outcomes.

This combined approach will also identify research gaps within the literature and form part of a descriptive narrative synthesis, supporting a broader understanding of the last two decades’ literature in this field.

The results will be reported in full by all authors and presented in accordance with the PRISMA-ScR checklist.^25 27 29^

All modifications to the search strategy and data extraction tool will be reported within the existing protocol registry on the OSF and in future publications.^25 29^

### Patient and public involvement

No patients or members of the public are involved in the scoping review.

## Ethics and dissemination

This scoping review will analyse existing published data and will not include the collection of data from human participants. Therefore, ethical approval is not required. However, the authors of this scoping review have complied with ethical guidance and standards of managing and reporting secondary data.

The findings of this review will be widely disseminated to maximise its impact and reach to those in the field and beyond. The findings will be shared through oral, visual and written formats that include oral and poster presentations at local, national and international conferences. In addition to submission to academic peer-reviewed journals for publication.

## Supplementary material

10.1136/bmjopen-2025-098745 online supplemental file 1

10.1136/bmjopen-2025-098745 online supplemental file 2
