# Supplementary material for: Role and responsibilities of a forensic mental health nurse: a scoping review protocol
Source: BMJ Open. 2025 Jul 15;15(7):e098745. doi: 10.1136/bmjopen-2025-098745 (PMC12265835; doi:10.1136/bmjopen-2025-098745)
Supplement: online supplemental file 1 [file bmjopen-15-7-s001.docx]

# **APPENDIX 1: SEARCH STRATEGY**

This search was constructed in the MEDLINE (EBSCO) database during May 2025 by the lead author and an Information Specialist/Librarian.

S1 (MH "Codes of Ethics") OR (MH "Ethics, Nursing") OR (MH "Ethics, Professional") OR (MH "Ethics, Clinical") OR (MH "Evidence-Based Nursing") OR (MH "Evidence-Based Practice") OR (MH "Evidence-Based Medicine") OR (MH "Nurse's Role")

S2 XB (((role* OR job OR employ* OR professional* OR workforce*) N3 (description* OR profil* OR competenc* OR skill* OR expect* OR perform* OR defin* OR boundaries OR clari* OR ambiguity OR conflict* OR clinical OR function* OR responsibilit* OR duty OR duties OR task* OR guideline* OR framework* OR regulat* OR registr* OR licensure OR accredit* OR scope OR profile OR identit* OR delineation OR boundar* OR statutory OR practice OR regulat* OR governance OR legislat* OR law OR policies OR policy OR standard*)) OR (workforce N3 plan*))

S3 XB (((performance OR practice OR professional OR regulatory OR workforce OR service* OR role* OR job) N3 (clarity OR chang* OR transition OR scope OR transformation OR innovat* OR adaptation OR qualif* OR assess* OR apprais* OR audit OR benchmarking OR evaluation OR accountability OR standard* OR progression OR evolution OR development OR regulation OR governance OR guidelines OR competencies)) OR "evidence-based practice" OR "code of practice" OR (continuing N3 education)))

S4 XB (("scope of practice" OR skillset* OR (skill* N3 set*) OR (decision* N3 making) OR "autonomy in practice" OR (care N3 deliver*) OR (case N3 manage*)))

S5 XB (((risk* N3 (plan* OR assess* OR protocol* OR evaluat* OR monitor* OR manag* OR report* OR document*)) OR "duty of care" OR ethic* OR (patient* N3 (advoc* OR right* OR consent)) OR (capacity N3 assessment*)))

S6 XB ((quality N3 improve*) OR audit* OR protocol* OR policy OR policies OR (staff N3 (training OR develop*)))

S7 S1 OR S2 OR S3 OR S4 OR S5 OR S6

S8 (MH "Forensic Nursing") OR (MH "Psychiatric Nursing")

S9 XB ((forensic* OR psychiat* OR inmate* OR incarcerat* OR correctional OR detain* OR custod* OR prisoner* OR (mental N2 health)) N3 nurs*))

S10 S8 OR S9

S11 (MH "Forensic Medicine+") OR (MH "Forensic Psychiatry") OR (MH "Health Services for Prisoners") OR (MH "Mental Disorders+/NU")

S12 (MH "Correctional Facilities+")

S13 XB ((forensic OR secur* OR custod* OR court* OR prison OR prisons OR hospital* OR ((low OR medium OR high) N3 (unit* OR setting*)) OR inpatient*) OR (disord* N3 offender*))

S14 S11 OR S12 OR S13

S15 S7 AND S10 AND S14
