# Supplementary material for: Role and responsibilities of a forensic mental health nurse: a scoping review protocol
Source: BMJ Open. 2025 Jul 15;15(7):e098745. doi: 10.1136/bmjopen-2025-098745 (PMC12265835; doi:10.1136/bmjopen-2025-098745)
Supplement: online supplemental file 2 [file bmjopen-15-7-s002.docx]

### **Appendix 2: DATA EXTRACTION FORM**

| **Category** | **Data to be Extracted** |
| --- | --- |
| **Citation** | Author  Title  Year of Publication |
| **Country of Origin** | Location country |
| **Research Summary** | Aims  Objectives  Setting |
| **Methodology** | Participants  Methodology |
| **Intervention** | Descriptive definition of role and responsibilities  Identified professional standards of practice influencing the role  Identified examples of good practice- this will include implicit and explicit examples |
| **Key results** | Outcomes/impact/results |
| **Reviewer comments** | Gaps in research  Reported limitations |
