# Supplementary material for: Role and responsibilities of a forensic mental health nurse: a scoping review protocol
Source: BMJ Open. 2025 Jul 15;15(7):e098745. doi: 10.1136/bmjopen-2025-098745 (PMC12265835; doi:10.1136/bmjopen-2025-098745)
Supplement: Supplementary file 3 [file bmjopen-2025-098745.reviewer_comments.pdf]

PEER REVIEW HISTORY

BMJ Open publishes all reviews undertaken for accepted manuscripts. Reviewers are asked to complete a checklist review form and are provided with free text boxes to elaborate on their assessment. These free text comments are reproduced below.

ARTICLE DETAILS

Title (Provisional)

The Role and Responsibilities of a Forensic Mental Health Nurse: A Systematic Scoping Review Protocol

Authors

Tulloch, Lindsay; Harkess-Murphy, EIleen; Walker, Helen; Cheyne, Joshua; McCaig, Marie; Ion, Robin

---

VERSION 1 - REVIEW

**Reviewer**	**1**
**Name**	**Barr, Lesley**
**Affiliation**	
**Date**	**09-Apr-2025**
**COI**	**None**

Thank you for the opportunity to review this manuscript. The subject of forensic mental health nursing roles and responsibilities is an important and evolving area that warrants further review. The protocol and objectives of the work are clearly defined.

I hope the following feedback is helpful in enhancing the manuscript:

- Abstract – ‘there has been significant growth in forensic mental health nursing (FMHN)’. What growth are you referring to? Increase scope of practice, nursing workforce or something else?

- Abstract – ‘Numerous scholars have explored the role definition and its complexities. However, the literature is dated and not relevant to the role and responsibilities of a forensic mental health nurse’. This is unclear. Why is the work of the previous scholars not relevant?

- Abstract – ‘This paper maps the current literature’. It’s more accurate to say that your paper maps the literature over the last 20 years (the date range of your search).

- Page 4, line 42 (Methods and Analysis). For the eligibility sentence, you state ‘setting’ but have not specified which setting (i.e prisons, courts, in-patient settings or community).

- Page 4, line 43, ‘All extracted literature will be exported into Endnote 21 in order to support the removal of duplicates, screening and selection process’. Consider ‘All extracted literature will be exported into Endnote 21 in order to support the removal of duplicates, [and assist in the] screening and selection process.

- Page 5 – keywords – I would recommend including the setting as a keyword (i.e. in-patient or prison).

- Page 5 – ‘collaborated with an experienced academic support from an Information Specialist/Librarian to develop the search strategy’ – this needs to be stated more clearly. Do you mean ‘Information Specialist/Library’ or ‘collaborated with an experienced academic Information Specialist/Librarian’.

- Page 5 – ‘for a broad and varied overview’. I am not clear what you mean by varied overview. Isn’t it a strength that you will be exploring the literature worldwide to identify variations in role definition?

- Page 6 (Introduction) –‘The role of a mental health nurse has grown considerably’. As mentioned before, grown in what way? Workforce numbers, scope of the role?

- Page 6 (Introduction) – The opening paragraph is confusing as it is discussing ‘mental health’ and ‘acute mental health’ nurses, when this manuscript is about forensic mental health nurses. The relevance is not clear.

- Page 6 – ‘They support patients in their recovery journey through a comprehensive rehabilitation programme of work’. Are you referring to occupational work, as in jobs? If not, Consider deleting ‘of work’.

- Page 7 – ‘The daily role of a FMHN will often include crisis management, identification of triggers, resulting in verbal de-escalation, and at times the implementation of interventions to ensure the protection of safety of others is maintained’. This needs to be made clearer.

- You have already mentioned in the abstract that scholars have already examined the role of FMHNs, and now you are discussing and using references to describe the role of forensic mental health nurses, so you need to clearly state why there is a need to conduct this scoping review. You have mentioned already that the literature is dated, which is a valid point, but what has changed in the FMHN landscape that makes this scoping review important. Consider things like changes in legislation that increase the population of prisoners with mental health issues, or a growing workforce within the FMHN specialty, or changes in university nurse training in the UK moving to more generic training content and what this means for emerging FMHNs. Also, nursing is now a very mobile workforce/career with nurses changing jobs frequently and moving across/within countries, which provides more imperative to standardise the definition of the FMHN role and responsibilities (for quality of care).

- Page 7 – ‘restrictive practices such as chemical, physical, and environmental restraint’. What about the use of seclusion (or is this what you are referring to with ‘environmental

restraint')? Whilst some countries no longer can use seclusion, it remains a defined restrictive practice globally.

- Page 7 – 'However much of the existing literature is almost two decades old'. There is a systematic literature review on the 'Roles and Responsibility of Forensic Psychiatric Nurse', which was completed in 2017 (10.14744/phd.2017.86094). This systematic review concluded that 'there were 'conflicts about their roles in units they work and that their roles were not clear'.

- Page 8 – 'Since this publication, much of the literature has been focused on its complexities'. What complexities do you mean? This isn't clear.

- Page 8 – 'guide nursing practice within forensic mental health settings are rooted within The Code professional standards of practice and behaviour for nurses, midwives and nursing associates'. Isn't this code generic across all nurses and midwives? Is there anything specific to FMHN? Make the case why this 'Code' is inadequate for FMHN.

- Page 9 – Methods – Can you state what the 5 step framework of Arksey and O'Malley's (2005) is (briefly).

- Page 9 – review questions. Is there a need to ask a question about nursing qualifications or training to be called a FMHN?

- Page 10 – Eligibility criteria (participants) -what about Enrolled Nurses? They are still in the workforce globally and will be reported in studies. Will this be a problem for you if you are only reporting on RNs. Will you be able to separate the data pertaining to RN and ENS if you only want to report on RNs?

- Page 10 – Context. What about courts? Many FMHNs work in court diversion programmes.

Best wishes in your research journey!

Reviewer 2

Name **Shetty, Shobha**

Affiliation **South East Technological University, Department of Nursing and Healthcare**

Date **14-Apr-2025**

COI **None**

Dear Authors

A much-needed exploration of current roles and responsibilities of mental health nurses working in forensic secure settings. My observation of your protocol are as follows;

Forensic mental health and FMH nursing are terms not known to all readers internationally. Therefore, introduce the reader to the forensic mental health service and a perspective on

mental health nurses working in these settings. Not all readers are familiar with the forensic mental health services.

Forensic mental health nursing (FMHN) is also referred to forensic mental health nurses in this article. Clearly identify these two terms for clarity.

Who are forensic mental health nurses? Is this term referring to the nurses working in forensic mental health services or is there a separate division they are registered as FMH nurses? If not, recognise that in the article.

Page 5- Introduction- 2nd paragraph- Explain relational security.

Page 8- Last paragraph- Says 'The formal search will commence in November 2024' while it is written in the abstract that the commencement of search is in December 2024. This also suggests that the review has already commenced. Has there been a delay in commencing this review?

Pay attention to grammatical/typo errors.

Page 9- Eligibility Criteria- Participants- are these nurses registered as mental health nurses or registered general nurses working in mental health settings?

Context- Are forensic child and adolescent mental health services included?

Publication criteria date range- Explain Why is this limited to 2024; we have already crossed the first quarter of 2025. Would articles published in 2025 excluded from this review? If so, the reason for exclusion.

Exclusion criteria 4 – Explain what is none adult forensic mental health settings.

Page 11-Data Extraction and Evidence selection- Are you using any web-based tool such as Covidence for Screening? If so, this is not evident in the article.

Explain Data extraction in detail- Will there be a pilot and any modification done to the data extraction tool?

Page 11- Data Analysis and presentation of results- Second paragraph- do you mean quantitative data?

Is qualitative data presented thematically? Explain.

Would PRISMA flow chart be used to report the results?

VERSION 1 - AUTHOR RESPONSE

Reviewer 1 Comments		Action Taken	Pg Num
Abstract	there has been significant growth in forensic mental health nursing (FMHN)'. What growth are you referring to? Increase	Changed to 'Over the past two decades FMHN has evolved	Pg 1

	scope of practice, nursing workforce or something else?	beyond custodial and containment practice. Contemporary…’	
	‘Numerous scholars have explored the role definition and its complexities. However, the literature is dated and not relevant to the role and responsibilities of a forensic mental health nurse’. This is unclear. Why is the work of the previous scholars not relevant?	Removed relevant and changed to read “Numerous scholars have examined the role of the FMHN………. mental health nurse relevant to contemporary FMHN practice.	Pg 1
	‘This paper maps the current literature’. It’s more accurate to say that your paper maps the literature over the last 20 years (the date range of your search).	Changed to read “This paper maps the literature over the last 20 years to establish…”	Pg 1
	Page 4, line 42 (Methods and Analysis). For the eligibility sentence, you state ‘setting’ but have not specified which setting (i.e prisons, courts, in-patient settings or community).	Changed to “forensic mental health nurse population and secure in-patient settings”.	Pg 3
	‘All extracted literature will be exported into Endnote 21 in order to support the removal of duplicates, screening and selection process’. Consider ‘All extracted literature will be exported into Endnote 21 in order to support the removal of duplicates, [and assist in the] screening and selection process.	Change to “All extracted literature will be exported into Endnote 21 in order to support the removal of duplicates, and assist in the screening and selection process”.	Pg 1
Key words	I would recommend including the setting as a keyword (i.e. in-patient or prison).	Inserted “secure in-patient”	Pg 2
	‘collaborated with an experienced academic support from an Information Specialist/Librarian to develop the search strategy’ – this needs to be stated more clearly. Do you mean ‘Information Specialist/Library’ or ‘collaborated with an experienced academic Information Specialist/Librarian’.	Removed “support from an”	Pg 3
	for a broad and varied overview’. I am not clear what you mean by varied overview. Isn’t it a strength that you will be exploring the literature worldwide to identify variations in role definition?	Changed to “Conducting a scoping review to explore…”	Pg 3
Intro	‘The role of a mental health nurse has grown considerably’. As mentioned before,	Changed to ‘In addition to the expanding scope of the workforce….’	Pg 6

	grown in what way? Workforce numbers, scope of the role?		
	The opening paragraph is confusing as it is discussing 'mental health' and 'acute mental health' nurses, when this manuscript is about forensic mental health nurses. The relevance is not clear.	Changed the opening paragraph to make clearer to the reader- "Forensic mental health nursing (FMHN) is a subspeciality....criminal justice systems"	Pg 1 & pg 4
	They support patients in their recovery journey through a comprehensive rehabilitation programme of work'. Are you referring to occupational work, as in jobs? If not, Consider deleting 'of work	Removed and amended to read 'FMHNs perform a wide range of clinical....'	Pg 4
	The daily role of a FMHN will often include crisis management, identification of triggers, resulting in verbal de-escalation, and at times the implementation of interventions to ensure the protection of safety of others is maintained'. This needs to be made clearer.<br><br>- You have already mentioned in the abstract that scholars have already examined the role of FMHNs, and now you are discussing and using references to describe the role of forensic mental health nurses, so you need to clearly state why there is a need to conduct this scoping review. You have mentioned already that the literature is dated, which is a valid point, but what has changed in the FMHN landscape that makes this scoping review important. Consider things like changes in legislation that increase the population of prisoners with mental health issues, or a growing workforce within the FMHN specialty, or changes in university nurse training in the UK moving to more generic training content and what this means for emerging FMHNs. Also, nursing is now a very mobile workforce/career with nurses changing jobs frequently and moving across/within countries, which provides more imperative to standardise	Amended and expanded to make clearer to the reader, "The daily role of a FMHN... applying restrictive practices as a last resort.<br><br>Added headings 'Knowledge Gap' and 'Educational Gap' to make clearer to the reader what has changed and why a scoping review is of importance now.	Pg 5<br><br>Pg 6 & Pg 7

	the definition of the FMHN role and responsibilities (for quality of care).		
	restrictive practices such as chemical, physical, and environmental restraint'. What about the use of seclusion (or is this what you are referring to with 'environmental restraint')? Whilst some countries no longer can use seclusion, it remains a defined restrictive practice globally.	Expanded and amended to read "This often necessitates the use of restrictive practices such as chemical, physical, and environmental restraint such as seclusion.....practices"	Pg 5
	However much of the existing literature is almost two decades old'. There is a systematic literature review on the 'Roles and Responsibility of Forensic Psychiatric Nurse', which was completed in 2017 (10.14744/phd.2017.86094). This systematic review concluded that 'there were 'conflicts about their roles in units they work and that their roles were not clear'	Amended to acknowledge and reference this paper, including the international ambiguity and conflict surrounding the role and responsibilities of the FMHN.	Pg 6
	Since this publication, much of the literature has been focused on its complexities'. What complexities do you mean? This isn't clear.	Expanded more to include 'such as the management of violence and aggression and restrictive practice use, recruitment and retention in FMHN'	Pg 6
	guide nursing practice within forensic mental health settings are rooted within The Code professional standards of practice and behaviour for nurses, midwives and nursing associates'. Isn't this code generic across all nurses and midwives? Is there anything specific to FMHN? Make the case why this 'Code' is inadequate for FMHN.	Amended and replaced with section on 'Educational Gaps'	Pg 7
Methods	Can you state what the 5 step framework of Arksey and O'Malley's (2005) is (briefly).	Inserted "Arksey and O'Malley's (2005) five step framework, which is a systematic approach to mapping literature concepts, evidence and gaps".	Pg 8

Review Questions	Is there a need to ask a question about nursing qualifications or training to be called a FMHN?	Added 'registered' to the review questions and refined the eligibility criteria.	Pg 8 & pg 9
Eligibility Criteria	what about Enrolled Nurses? They are still in the workforce globally and will be reported in studies. Will this be a problem for you if you are only reporting on RNs. Will you be able to separate the data pertaining to RN and ENS if you only want to report on RNs?	Changed eligibility criteria- 'Participants: registered mental health nurses..'<br><br>Changed exclusion criteria-<br>'Research conducted on forensic mental health professionals other than registered....	Pg 9<br><br>Pg 9 & 10
Context	What about courts? Many FMHNs work in court diversion programmes.	Amended to include secure in-patient, court and prison.	Pg 9
**Reviewer 2 Comments**		**Action Taken**	**Pg Num**
	Forensic mental health and FMH nursing are terms not known to all readers internationally. Therefore, introduce the reader to the forensic mental health service and a perspective on mental health nurses working in these settings. Not all readers are familiar with the forensic mental health services.<br>Forensic mental health nursing (FMHN) is also referred to forensic mental health nurses in this article. Clearly identify these two terms for clarity.<br>Who are forensic mental health nurses? Is	Amended to explain what forensic mental health nursing is in the;<br><br>abstract "Forensic mental health nursing (FMHN) is a subspeciality of psychiatric nursing.... systems.<br><br>Introduction "Forensic mental health nursing	Pg 1<br><br>Pg 4

	this term referring to the nurses working in forensic mental health services or is there a separate division they are registered as FMH nurses? If not, recognise that in the article.	care are.....and/or others.	
Intro	Explain relational security	Amended to read "Relational security is a key component in forensic services. It refers to...'	Pg 4, Pg 5
	The formal search will commence in November 2024' while it is written in the abstract that the commencement of search is in December 2024. This also suggests that the review has already commenced. Has there been a delay in commencing this review?<br><br>Pay attention to grammatical/typo errors.	Yes- there has been a delay due to competing demands. Amended to read, 'Publication criteria date range between 2004-and first quarter of 2025.' Reviewed the paper to checked for errors.	Abstract- pg 1<br><br>Eligibility criteria- pg 9<br><br>Full protocol review
Elig Criteria	Participants- are these nurses registered as mental health nurses or registered general nurses working in mental health settings?<br><br>Context- Are forensic child and adolescent mental health services included?	Amended eligibility and exclusion criteria to read 'registered mental health nurses practicing within a forensic mental health setting'<br><br>No, adult only. Amended eligibility and exclusion	Pg 9 & pg 10<br><br>Pg 9 & pg 10
	Publication criteria date range- Explain Why is this limited to 2024; we have already crossed the first quarter of 2025. Would articles published in 2025 excluded from this review? If so, the reason for exclusion.	There was a delay in commencing the review- amended to read, "Publication criteria date range between 2004-2024 including first quarter of 2025."	Pg 1, Pg 9
Excluded criteria	Explain what is none adult forensic mental health settings	Inserted "such as child, adolescent or geriatric forensic mental health"	Pg 9
Data Extraction	Are you using any web-based tool such as Covidence for Screening? If so, this is not	No. Amended data extraction process & data analysis and	Pg 11 & pg 12

	evident in the article.<br><br><br><br><br>Explain Data extraction in detail- Will there be a pilot and any modification done to the data extraction tool?	presentation of results sections to reference which author(s) will carry out specific stages of the screening.<br><br>Amended to add pilot of data extraction tool, including where it would be reported.	Pg 12
Data Analysis	do you mean quantitative data?	Amended to read quantitative.	Pg 12
Pres of results	Is qualitative data presented thematically? Explain.	Amended and expanded to explain further. 'Qualitative findings will be synthesised using thematic analysis…'	Pg 12
	Would PRISMA flow chart be used to report the results?	Yes, I have moved this from methods and analysis section to data analysis and presentation of results section.	Pg 11 & pg 12
